# MicroRNA *miR-378* promotes BMP2-induced osteogenic differentiation of mesenchymal progenitor cells

**DOI:** 10.1186/1471-2199-15-1

**Published:** 2014-01-27

**Authors:** Marlinda Hupkes, Ana M Sotoca, José M Hendriks, Everardus J van Zoelen, Koen J Dechering

**Affiliations:** 1 Department of Cell & Applied Biology, Faculty of Science, Nijmegen Centre for Molecular Life Sciences (NCMLS), Radboud University Nijmegen, Heyendaalseweg 135, 6525 AJ, Nijmegen, The Netherlands; 2 Merck Research Laboratories, PO Box 20, 5340 BH, Oss, The Netherlands; 3 Current affiliation: TropIQ Health Sciences, PO Box 9101, 6500 HB, Nijmegen, The Netherlands

## Abstract

**Background:**

MicroRNAs (miRNAs) are a family of small, non-coding single-stranded RNA molecules involved in post-transcriptional regulation of gene expression. As such, they are believed to play a role in regulating the step-wise changes in gene expression patterns that occur during cell fate specification of multipotent stem cells. Here, we have studied whether terminal differentiation of C2C12 myoblasts is indeed controlled by lineage-specific changes in miRNA expression.

**Results:**

Using a previously generated RNA polymerase II (Pol-II) ChIP-on-chip dataset, we show differential Pol-II occupancy at the promoter regions of six miRNAs during C2C12 myogenic versus BMP2-induced osteogenic differentiation. Overexpression of one of these miRNAs, miR-378, enhances Alp activity, calcium deposition and mRNA expression of osteogenic marker genes in the presence of BMP2.

**Conclusions:**

Our results demonstrate a previously unknown role for miR-378 in promoting BMP2-induced osteogenic differentiation.

## Background

The generation of distinct populations of terminally differentiated, mature specialized cell types from multipotent stem cells, via progenitor cells, is characterized by a progressive restriction of differentiation potential that involves a tightly controlled, coordinated activation and repression of specific subsets of genes. This process depends on the orchestrated action of key regulatory transcription factors in combination with changes in epigenetic modifications that regulate which regions in the genome are accessible for transcription [1]. The more recently discovered family of microRNAs (miRNAs) is thought to provide an additional layer of gene control that integrates with these transcriptional and epigenetic regulatory processes to further modulate the final gene expression profile of a specific cell type [2].

MicroRNAs (miRNAs) are a class of small, evolutionarily conserved non-coding RNA molecules (~19-25 nucleotides) involved in post-transcriptional gene silencing and as such play important roles in diverse biological processes such as developmental timing [3], insulin secretion [4], apoptosis [5], oncogenesis [6] and organ development [7,8]. MiRNAs are transcribed from the genome as long primary transcripts (pri-miRNA) encoding one or more miRNAs, which are processed in the nucleus by the so-called ‘microprocessor’ complex consisting of DGCR8 (DiGeorge Syndrome Critical Region 8) and the ribonuclease III (RNase III) enzyme DROSHA [9]. This liberates the precursor-miRNA (pre-miRNA), a hairpin-type structure, which has a characteristic 3’ overhang of two nucleotides and is subsequently exported from the nucleus by Exportin-5, a RAN GTPase protein [10]. Inside the cytoplasm, the pre-miRNA hairpin loop is removed by a second RNase III enzyme, DICER, yielding a ~22 nucleotide long imperfect RNA duplex. This duplex contains two potentially functional mature miRNAs termed the 5p and 3p strands, referring to which end of the pre-miRNA they are derived from [8]. One of these strands is then incorporated into the RNA-induced silencing complex (RISC), which guides the mature miRNA to its target mRNA. In general, one strand is inserted into the RISC complex at much higher efficiency than the other, whereby the strand choice may be based on a variety of factors including thermodynamic instability, strength of the base-pairing and position of the stem-loop [11]. The strand that is incorporated into RISC with lowest efficiency is referred to with an asterisk (miRNA*) and, since non-incorporated strands are thought to be degraded, is less-abundant than its counterpart [12].

The RISC-incorporated miRNA regulates gene expression through sequence-specific interactions with its target site, which is typically located within the 3’ untranslated region (3’UTR) of an mRNA transcript. Animal miRNAs usually exhibit only partial complementarity to their mRNA targets, whereby nucleotides 2–8 at the 5’ end of the miRNA, referred to as the ‘seed region’, are thought to be the primary determinant of target specificity [11,13]. Interaction of the miRNA with its target mRNA can interfere with protein translation and/or induce mRNA degradation through a variety of different mechanisms [14], thereby decreasing the protein output. The mechanism and level of effect are thought to be influenced by the degree of complementarity between the miRNA and its mRNA target, the surrounding sequences in the target 3’UTR [12] and their relative abundance [15].

Estimated numbers of miRNA genes amount to nearly 1% of the number of predicted protein-coding genes in the genome of higher eukaryotes, a percentage similar to that of other large gene families with regulatory roles, such as the homeodomain transcription factor family [11]. In addition, miRNAs are estimated to target the expression of approximately one-third of all mammalian genes [8]. Due to the imperfect complementarity between a miRNA and its target, most miRNAs are predicted to be able to bind to and regulate a large number of different mRNA targets [2]. In addition, multiple different miRNAs can synergistically target and control a single mRNA target [2], providing the potential for complex regulatory networks. Many miRNAs studied so far are differentially expressed during development and differentiation, suggesting that each cell type might have its own unique miRNA profile that could affect the utilization of thousands of mRNAs and thus ‘micromanage’ the output of the transcriptome [2,8]. Several studies have indeed provided examples of miRNAs that play a role in the regulation of cellular differentiation, including hematopoietic cell differentiation [16], adipogenesis [17], osteogenesis [18] and myogenesis [12]. In addition, it has been shown that expression of only three miRNAs (*miR-200c, miR-302* and *miR-369*) is sufficient to induce pluripotency in mouse cells, demonstrating that miRNAs can act as major determinants of cell fate [19]. Since miRNAs have been discovered relatively recently, however, much still remains to be learned about their role in cellular programming, including the identification and detailed analysis of their targets.

In the present study, we took advantage of the robust and homogeneous differentiation characteristics of the mouse C2C12 myoblast cell line to investigate whether lineage-specific changes in miRNA expression might underlie their terminal differentiation. C2C12 cells were originally derived from regenerating muscle tissue [20] and are considered to represent the transit amplifying progenitor population that is derived from muscle satellite stem cells [21]. When cultured under low-serum conditions, C2C12 cells terminally differentiate and fuse into multi-nucleated myotubes upon reaching confluence, which is preceded by upregulation of the key myogenic transcription factors *Myod1* and *Myog*. However, treatment of C2C12 cells with bone morphogenetic protein (BMP) 2 induces these cells to differentiate into osteoblasts, which involves the upregulation of key osteogenic transcription factors *Dlx5*, *Sp7* and *Runx2*[22-24], subsequently leading to the expression of late osteoblast marker genes, such as *Alpl* and *Bglap*[25,26]. These characteristics make C2C12 progenitor cells an excellent model system to study the molecular mechanisms that underlie cell-fate specification and terminal differentiation. Using a previously generated RNA polymerase II (Pol-II) ChIP-on-chip dataset [27], we show that several miRNAs have differential Pol-II occupancy during C2C12 myogenic versus osteogenic differentiation and that overexpression of one of these miRNAs, miR-378, promotes BMP2-induced osteogenic differentiation of C2C12 cells.

## Results

### C2C12 lineage-specific miRNA expression

**Figure 1:**
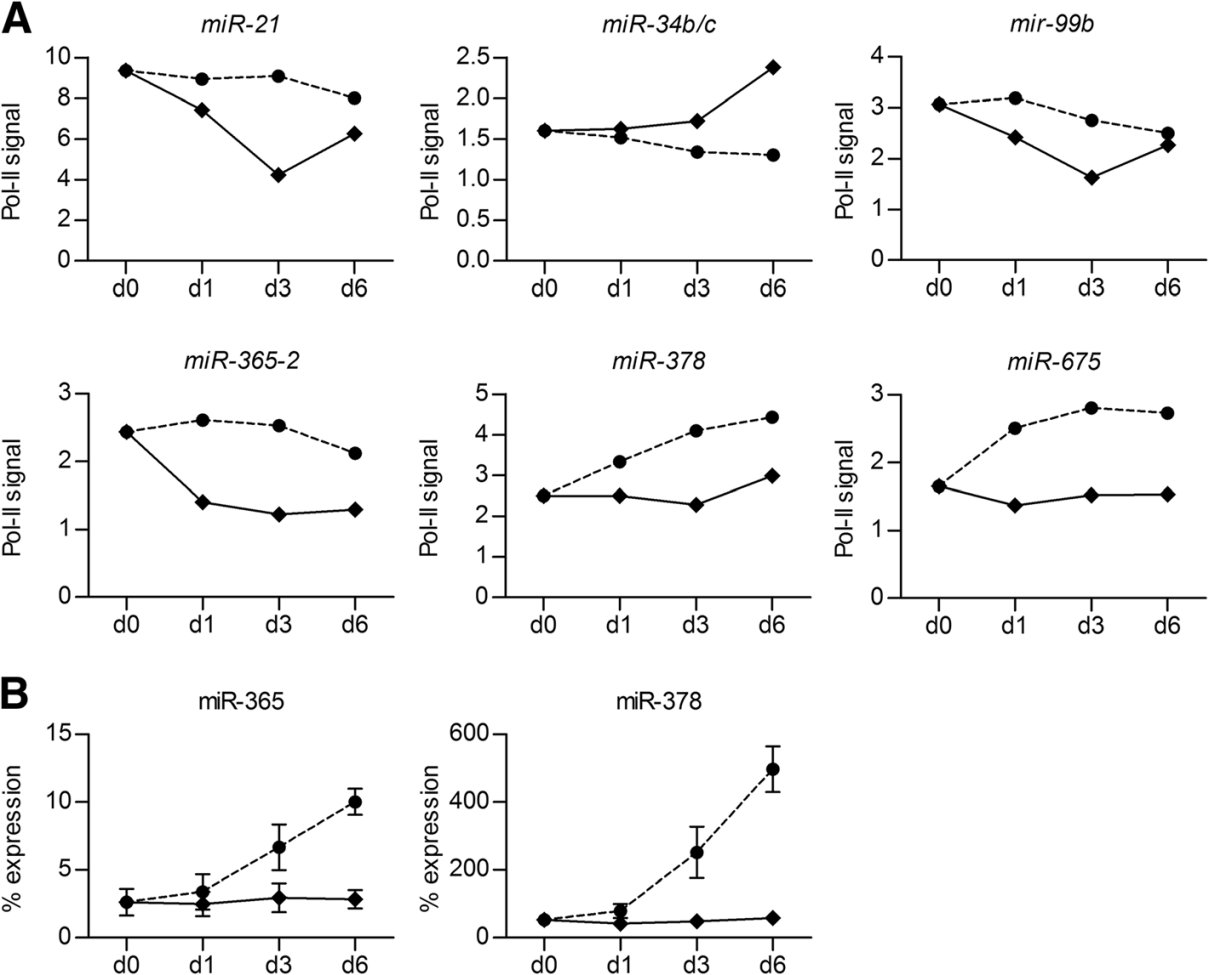
**C2C12 lineage-specific miRNA expression.** C2C12 cells were treated with (diamonds) or without (circles) 300 ng/ml BMP2 for 6 days, during which chromatin and RNA were harvested in parallel at d0, d1, d3 and d6. **A)** Pol-II enrichment [27] at regions associated with miRNA genes *miR-21* (RefSeq Accession NR_029738; enriched region from −1492 to +1661 bp), *miR-34b/c* (RefSeq Accessions NR_029655 and NR_029654; enriched regions from −287 to +236 bp (*miR-34b*) and −822 to −299 bp (*miR-34c*)), *miR-99b* (RefSeq Accession NR_029536; enriched region from −1113 to −399 bp), *miR-365-2* (RefSeq Accession NR_029959; enriched region from +187 to +1282 bp), *miR-378* (RefSeq Accession NR_029879.1; enriched region from −2068 to −130 bp) and *miR-675* (RefSeq Accession NR_030416.1; enriched region from −1055 to −235 bp) as determined by Pol-II ChIP-on-chip analysis of single biological samples [27]. **B)** Mature miRNA levels of *miR-365* (*3p*) and *miR-378* (*3p*) as determined by real-time PCR and expressed as a percentage of the control small, non-coding RNA *snoRNA202*. The mean values +/− SD from duplicate measurements are shown for all data points.

To identify miRNAs that are differentially expressed during C2C12 myogenic versus BMP2-induced osteogenic differentiation, and thereby might play a role in lineage restriction, we made use of our previously generated Pol-II ChIP-on-chip dataset [27]. This dataset contains Pol-II occupancy data for undifferentiated C2C12 cells (d0) and cells treated with (osteogenesis) or without (myogenesis) BMP2 for 1, 3 and 6 days, whereby changes in Pol-II occupancy are considered to reflect changes in transcriptional activity. Since miRNA genes are usually also regulated by Pol-II promoters [28], this dataset formed a good starting point to search for lineage-specific miRNA expression profiles. Our selection criteria (see Methods) thus led to the identification of 6 miRNA genes, namely *miR-21, miR-34b/c, miR-99b, miR-365-2, miR-378* and *miR-675*, located in the vicinity of enriched regions with differential Pol-II occupancy profiles during myogenic versus osteogenic differentiation within our dataset (Figure 1A).

Since most of these enriched Pol-II regions could alternatively be associated to other surrounding (predicted) genes, we subsequently validated whether the identified Pol-II occupancy profiles correspond to the actual expression profile of two of these miRNAs, *miR-365* and *miR-378*, by quantitative PCR analysis of the mature miRNAs (Figure 1B). For miR-365, the higher levels of Pol-II occupancy on the associated enriched region during myogenesis versus osteogenesis is reflected by higher levels of mature miRNA expression. While Pol-II occupancy appears to be specifically downregulated during osteogenesis and does not change during myogenesis, however, mature miR-365 levels do not change during osteogenesis and are upregulated during myogenesis. For miR-378, the associated Pol-II occupancy profile and the mature miRNA expression pattern are very similar. These results confirm a lineage-specific difference in the expression of both miR-365 and miR-378. Given the high expression levels of mature miR-378 relative to miR-365, we subsequently focused on this miRNA to further investigate its potential role in C2C12 lineage-specific differentiation.

### Effect of miR-378 overexpression on genome-wide mRNA expression levels

To gain more understanding of the role and putative target of miR-378 in C2C12 differentiation, we first created a stably transduced C2C12 cell line overexpressing miR-378 (C2C12-pMirn378) and a control cell line transduced with the parent vector (C2C12-pMirn0). We subsequently examined the effect of miR-378 overexpression on gene expression levels during C2C12 lineage-specific differentiation by means of genome-wide mRNA profiling of undifferentiated (d0) C2C12-pMirn378 and control C2C12-pMirn0 cells and of both cell lines treated with or without BMP2 for 3 and 6 days.

We first explored changes in gene expression levels during differentiation of the control C2C12-pMirn0 cells. Comparison of expression levels in differentiating cells (d3 and d6 time points) versus undifferentiated (d0) cells in this control group revealed a significant upregulation of 4521 probes during C2C12-pMirn0 treatment without BMP2. Functional gene annotation of this set of probes according to Gene Ontology (GO: biological processes category) revealed significant enrichment of many GO terms related to muscle development (including for example ‘muscle organ development’, ‘striated muscle cell development’, ‘muscle contraction’ and ‘muscle cell development’; data not shown), consistent with an upregulation of the muscle transcription program under these culture conditions. This is illustrated by the expression profiles of several myogenic marker genes in our control C2C12-pMirn0 cells (Additional file 1A).

Similarly, we observed a significant upregulation of 4664 probes during C2C12-pMirn0 treatment with BMP2 (d3 and d6 time points) as compared to undifferentiated (d0) cells in the control group. Functional gene annotation of these probes according to GO (biological processes) revealed significant enrichment of GO terms related to bone development (including ‘skeletal system development’, ‘bone development’, ‘extracellular matrix organization’ and ‘ossification’, ‘skeletal system morphogenesis’, ‘osteoblast differentiation’, ‘bone mineralization’; data not shown), consistent with the expected osteogenesis-inducing effect of BMP2 on our control C2C12-pMirn0 cells. The expression profiles of several osteogenic marker genes are presented in Additional file 1B.

Finally, control C2C12-pMirn0 cultures treated both with and without BMP2 showed a clear cell cycle withdrawal signature as common functional gene annotation of the sets of probes significantly downregulated during myogenic (5396 probes) and osteogenic (4550 probes) differentiation. To illustrate, the expression profiles of several cell-cycle regulators are shown in Additional file 1C.

We thus conclude that treatment of our control C2C12-pMirn0 cells with and without BMP2 had induced the expected changes in transcription patterns corresponding to osteogenic and myogenic differentiation, respectively.

**Figure 2:**
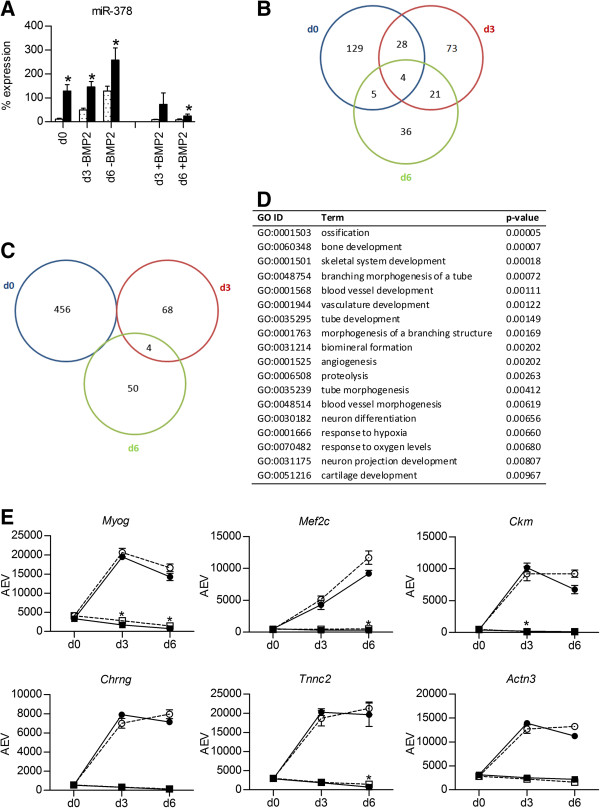
**Effect of miR-378 overexpression on C2C12 muscle transcription program. A)** Mature miR-378 levels in C2C12-pMirn0 (light bars) and C2C12-pMirn378 (dark bars) cells at indicated time points during treatment with or without 300 ng/ml BMP2. MiR-378 levels are expressed as a percentage of the control small, non-coding RNA *snoRNA202*. Mean values +/− SD of three biological replicates, whereby each measurement was made in duplicate, are shown. *p < 0.05 when compared to the C2C12-pMirn0 sample at the same time point and treatment. **B-C)** Venn diagrams representing the number of probes on the microarray that are significantly lower **(B)** or higher **(C)** expressed in C2C12-pMirn378 cells than in control C2C12-pMirn0 cells at each time point during differentiation in the absence of BMP2. **D)** Enriched (p < 0.01) GO terms within the set of probes that are significantly lower expressed in C2C12-pMirn378 versus C2C12-pMirn0 cells at at least two consecutive time points during differentiation in the absence of BMP2. **E)** mRNA expression profiles of the muscle transcription factors myogenin (*Myog;* 1419391_at) and myocyte enhancer factor 2C (*Mef2c;* 1421027_a_at) and other muscle marker genes muscle creatine kinase (*Ckm;* 1417614_at), the acetylcholine receptor subunit gamma (*Chrng;* 1449532_at) and the sarcomeric genes fast troponin C2 (*Tnnc2;* 1417464_at) and actinin alpha 3 (*Actn3;* 1418677_at) at indicated time points during differentiation of C2C12-pMirn0 (light bullets) and C2C12-pMirn378 (dark bullets) cells treated with (squares) or without (circles) 300 ng/ml BMP2 as revealed from microarray analysis. Mean expression values +/− SD from triplicate microarray experiments are shown for all data points. When the error bar is not visible, the SD falls within the printed data point. All SD values are, however, listed in Additional file 2. AEV = average expression value; *significantly different expression in C2C12-pMirn378 compared to control C2C12-pMirn0 at the same time point and treatment.

We next examined the effect of miR-378 overexpression on these gene expression profiles. MiR-378 is expressed approximately 11-fold higher in C2C12-pMirn378 cells than in C2C12-pMirn0 cells at the d0 time point (Figure 2A). Similar to C2C12-pMirn0 cells, miR-378 expression increases during myogenic differentiation of C2C12-pMirn378 cells (Figure 2A). While miR-378 levels remain higher in C2C12-pMirn378 versus C2C12-pMirn0 cells during myogenesis, the fold overexpression decreases to approximately 3-fold at d3 and 2-fold at d6 (Figure 2A). The fold overexpression of miR-378 in C2C12-pMirn378 versus C2C12-pMirn0 cells also decreases to approximately 8-fold at d3 and 3-fold at d6 during BMP2-induced osteogenesis (Figure 2A).

**Figure 3:**
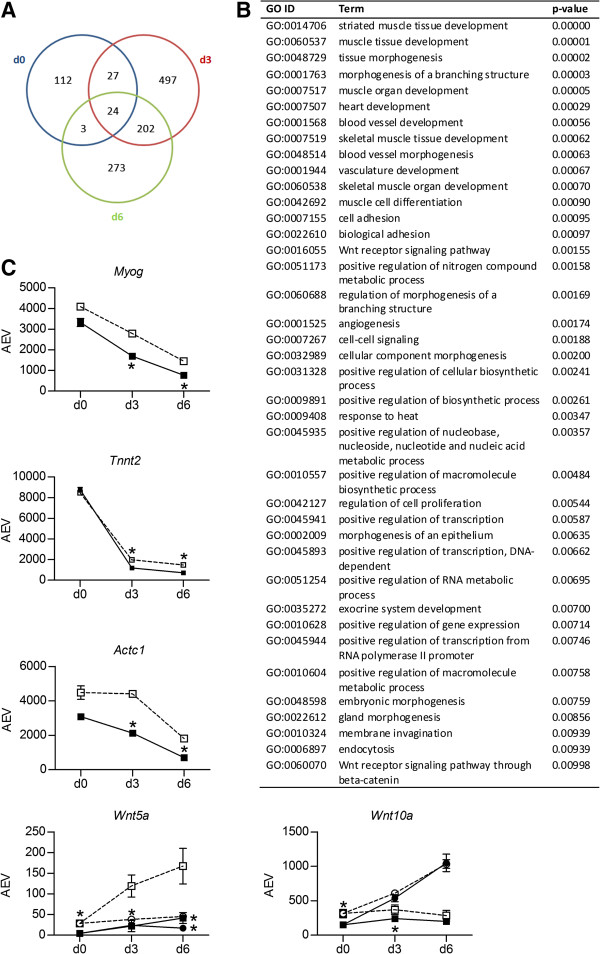
**Effect of miR-378 overexpression on C2C12 bone transcription program: downregulation. A)** Venn diagram representing the number of probes on the microarray that are significantly (as defined in Methods) lower expressed in C2C12-pMirn378 cells than in control C2C12-pMirn0 cells at each time point during differentiation in the presence of 300 ng/ml BMP2. **B)** Enriched (p < 0.01) GO terms (in biological processes category) within the set of probes that are significantly lower expressed in C2C12-pMirn378 versus C2C12-pMirn0 cells at at least two consecutive time points during differentiation in the presence of 300 ng/ml BMP2. **C)** Microarray profiles of representative genes that are expressed at significantly lower levels in C2C12-pMirn378 cells than in C2C12-pMirn0 cells at at least two consecutive time points during differentiation in the presence of BMP2 (light bullets: C2C12-pMirn0, dark bullets: C2C12-pMirn378, squares: treatment with BMP2, circles: treatment without BMP2); *Myog,* troponin T2, cardiac (*Tnnt2;* 1418726_a_at), actin alpha cardiac muscle 1 (*Actc1;* 1415927_at), wingless-related MMTV integration site 5a (*Wnt5a;* 1436791_at) and 10a *(Wnt10a;* 1460657_at). For *Myog, Tnnt2* and *Actc1*, expression levels are only shown for cells treated with BMP2. Mean expression values +/− SD from triplicate microarray experiments are shown for all data points. When the error bar is not visible, the SD falls within the printed data point. All SD values are, however, listed in Additional file 2. AEV = average expression value; *significantly different expression in C2C12-pMirn378 compared to control C2C12-pMirn0 at the same time point and treatment (see Methods for definition).

**Figure 4:**
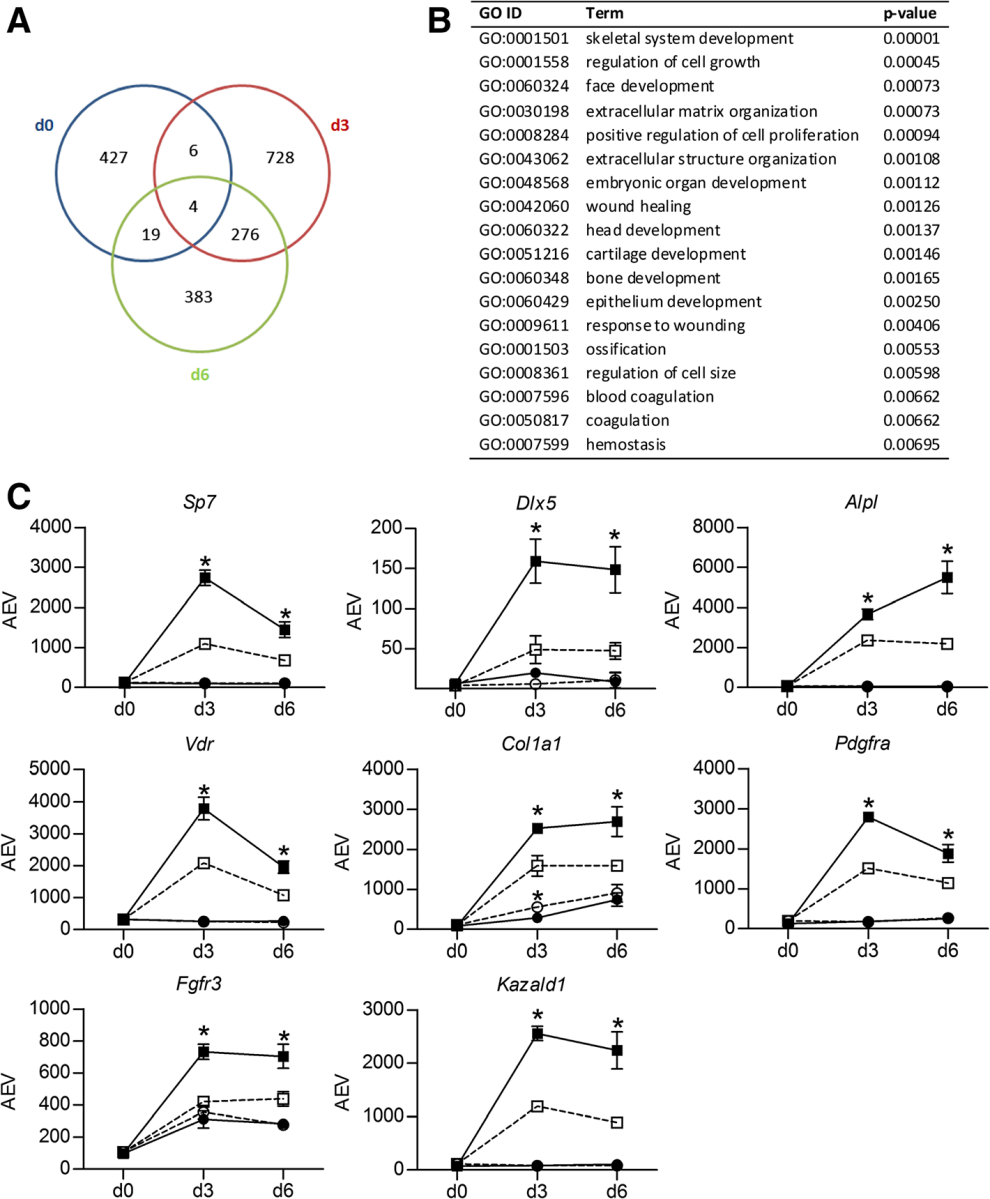
**Effect of miR-378 overexpression on C2C12 bone transcription program: upregulation. A)** Venn diagram representing the number of probes on the microarray that are significantly (as defined in Methods) higher expressed in C2C12-pMirn378 cells than in control C2C12-pMirn0 cells at each time point during differentiation in the presence of 300 ng/ml BMP2. **B)** Enriched (p < 0.01) GO terms (in biological processes category) within the set of probes that are significantly higher expressed in C2C12-pMirn378 versus C2C12-pMirn0 cells at at least two consecutive time points during differentiation in the presence of 300 ng/ml BMP2. **C)** Microarray profiles of bone markers Sp7 transcription factor 7 (*Sp7;* 1418425_at), distal-less homeobox 5 (*Dlx5;* 1449863_a_at)*,* alkaline phosphatase (*Alpl;* 1423611_at), vitamin D receptor (*Vdr;* 1418175_at)*,* collagen type I alpha 1 (*Col1a1;* 1423669_at)*,* platelet derived growth factor receptor alpha polypeptide (*Pdgfra;* 1421917_at)*,* fibroblast growth factor receptor 3 (*Fgfr3;* 1421841_at) and Kazal-type serine peptidase inhibitor domain 1 (*Kazald1;* 1436528_at) at indicated time points during differentiation of C2C12-pMirn0 (light bullets) and C2C12-pMirn378 (dark bullets) cells treated with (squares) or without (circles) 300 ng/ml BMP2 as revealed from microarray analysis. Mean expression values +/− SD from triplicate microarray experiments are shown for all data points. When the error bar is not visible, the SD falls within the printed data point. All SD values are, however, listed in Additional file 2. AEV = average expression value; *significantly different expression in C2C12-pMirn378 compared to control C2C12-pMirn0 at the same time point and treatment (see Methods for definition).

Gene expression levels in C2C12-pMirn378 cells were compared to those in control C2C12-pMirn0 cells for each time point during each treatment separately. The Venn diagrams in Figure 2B-C, Figure 3A and Figure 4A demonstrate the number of probes found to be significantly higher- or lower expressed in the C2C12-pMirn378 cells versus C2C12-pMirn0 cells at each indicated time point during myogenesis (Figure 2B-C) and osteogenesis (Figures 3A and 4A). We subsequently focused on the sets of probes that are consistently expressed at either higher or lower levels at at least two consecutive time points during differentiation.

The Venn diagram in Figure 2C shows that during myogenic differentiation hardly any probes are consistently higher expressed in C2C12-pMirn378 cells than in the C2C12-pMirn0 cells. However, we did observe a significantly lower expression of 53 probes at two or more consecutive time points (Figure 2B). GO-analysis of this set of probes (Figure 2D) revealed a significant enrichment of GO terms associated with various alternative differentiation pathways, including osteogenesis, blood vessel development, neuron differentiation and cartilage development. Most of these genes are, however, upregulated during (both C2C12-pMirn378 and C2C12-pMirn0) myogenic differentiation, so they do not appear to be specific for a particular lineage. We did not observe any significant differences between C2C12-pMirn378 and C2C12-pMirn0 cells in the expression of muscle marker genes, such as for example the myogenic transcription factors *Myog* and *Mef2c*, *Ckm*, *Chrng* and the sarcomeric proteins *Actn3* and *Tnnc2* during myogenesis (Figure 2E), suggesting that miR-378 overexpression does not have an effect on C2C12 muscle differentiation.

Compared to myogenesis, many more probes are differentially expressed in C2C12-pMirn378 cells versus C2C12-pMirn0 cells during osteogenic differentiation (Figure 3A and Figure 4A). We observed a consistent (at at least two consecutive time points) lower expression of 253 probes (Figure 3A) and higher expression of 286 probes (Figure 4A) in the C2C12-pMirn378 cells. GO-analysis showed that the set of lower expressed probes was significantly enriched for numerous GO terms associated with muscle differentiation (including for example ‘striated muscle development’, ‘muscle tissue development’ and ‘muscle organ development’: Figure 3B), and includes genes such as the myogenic transcription factor *Myog* and the sarcomeric proteins *Tnnt2* and *Actc1.* These genes, which are upregulated during (both C2C12-pMirn378 and C2C12-pMirn0) myogenesis, are downregulated during BMP2-induced osteogenesis of C2C12-pMirn0 cells, which is further enhanced in C2C12-pMirn378 cells (Figure 3C). Besides terms associated with muscle differentiation, GO-analysis also revealed significant enrichment of GO terms associated with Wnt signaling (‘Wnt receptor signaling pathway’ and ‘Wnt receptor signaling pathway through beta-catenin’), which include genes for the Wnt proteins *Wnt5a* and *Wnt10a*. In control C2C12-pMirn0 cells, *Wnt10a* is upregulated specifically during myogenesis, while *Wnt5a* is upregulated specifically during BMP2-induced osteogenesis (Figure 3C).

Interestingly, GO-analysis of the set of 286 probes that are consistently expressed higher in C2C12-pMirn378 cells than in C2C12-pMirn0 cells during BMP2 treatment revealed significant enrichment of GO terms related to bone differentiation (including ‘skeletal system development’, ‘extracellular matrix organization’, ‘bone development’ and ‘ossification’: Figure 4B), and includes genes for the osteogenic transcription factors *Sp7* and *Dlx5* and other osteogenic marker genes such as *Alpl, Vdr, Col1a1, Pdgfra, Fgfr3* and *Kazald1* (Figure 4C). The higher expression of osteogenic marker genes in C2C12-pMirn378 cells versus control C2C12-pMirn0 cells suggests that overexpression of miR-378 has a positive effect on C2C12 BMP2-induced osteogenic differentiation.

### Putative miR-378 target selection and validation

While our mRNA profiling analysis revealed that a large number of genes are affected by miR-378 overexpression, we expected the majority of these changes in expression to be the result of indirect, downstream events following the initial effect of miR-378 on its direct target(s). We therefore set out next to identify direct miR-378 target genes. Given the general effect of miR-378 overexpression on osteogenesis, we hypothesized that miR-378 might target signaling pathways involved in the initial activation of the osteogenic transcription program. We therefore focused on genes that were downregulated by miR-378 overexpression early during BMP2 treatment (i.e. at at least the d0 and d3 time point) and had at least one predicted miR-378 target site in their 3’UTR (see Methods). From this group, we selected three candidate target genes that are known to play a role in the regulation of osteoblast differentiation: the Wnt signaling proteins *Wnt5a* and *Wnt10a* and the BMP-inhibitor *Grem1* (gremlin 1).

To determine whether these candidates are indeed directly targeted by miR-378, we used an *in vitro* luciferase reporter assay. Reporter constructs containing the 3’UTRs of *Wnt5a, Wnt10a* and *Grem1*, as well as a positive control containing the miR-378 target sequence, fused to a luciferase reporter gene were co-transfected into HEK293 cells together with the miR-378 overexpression pMirn378 or control plasmid pMirn0 to examine changes in luciferase activity (see Methods). Overexpression of miR-378 significantly suppressed luciferase activity of the positive control, but had no significant effect on the 3’UTR-luciferase reporter constructs (data not shown). Our selected candidates therefore do not appear to be direct targets of miR-378.

### Effect of miR-378 overexpression on C2C12 differentiation

**Figure 5:**
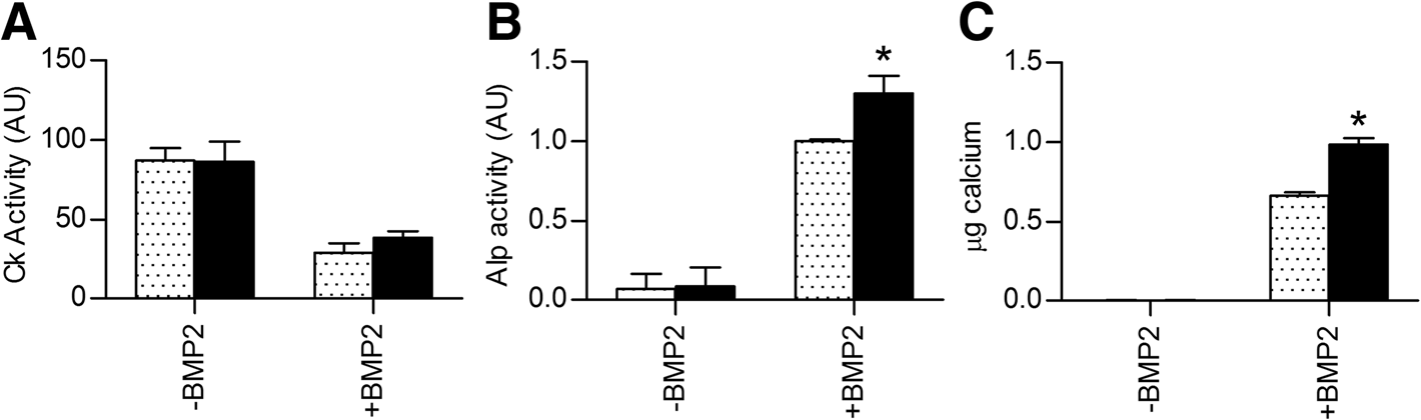
**Effect of miR-378 overexpression on C2C12 differentiation. A-C)** Creatine kinase **(A)** and Alp **(B)** activity in and calcium deposition **(C)** by C2C12-pMirn0 (light bars) and C2C12-pMirn378 (dark bars) cells after 6 **(A-B)** or 10 **(C)** days of culture in the presence or absence of 300 ng/ml BMP2. **A)** The mean values +/− SD of two biological replicates, whereby each measurement was made in duplicate, are shown. **B)** Mean values +/− SD of three independent experiments with biological duplicates each. For each independent experiment, Alp activity values were normalized to the average of the C2C12-pMirn0 samples treated with BMP2. **C)** Data shows calcium deposition in 24-well plates and is representative of 2 independent experiments. Mean values +/− SD of two biological replicates in one experiment, whereby each measurement was made in duplicate, are shown. *p < 0.05 when compared to the C2C12-pMirn0 sample with the same treatment. AU: arbitrary units.

Finally, we examined the overall effect of miR-378 overexpression on C2C12 myogenesis and osteogenesis by means of biochemical assays for differentiation markers. The effect on myogenic differentiation was assessed by comparing creatine kinase (Ck) activity in C2C12-pMirn0 and C2C12-pMirn378 cells after treatment with DM in the absence of BMP2 (Figure 5A). Consistent with the lack of effect on myogenic marker gene expression, no significant differences in Ck activity were observed between the two cell lines, again indicating that overexpression of miR-378 does not affect C2C12 myogenesis.

The effect of miR-378 overexpression on osteogenesis was assessed by comparing alkaline phosphatase (Alp) activity in and calcium release by C2C12-pMirn0 and C2C12-pMirn378 cells after treatment with BMP2 (Figure 5B-C). The results demonstrate both an increase in Alp activity and a significant enhancement of calcium deposition by the C2C12-pMirn378 cells. In agreement with the higher expression levels of osteogenic marker genes observed in this cell-line, these results further indicate that overexpression of miR-378 enhances C2C12 BMP2-induced osteogenesis.

## Discussion

In this study we used a previously generated Pol-II ChIP-on-chip dataset to identify miRNAs that are differentially expressed during C2C12 myogenic versus osteogenic differentiation and thus possibly play a role in lineage specification. Overexpression of one of these miRNAs, miR-378, had no apparent effect on myogenesis while enhancing BMP2-induced osteogenesis, suggesting a positive role for this miRNA in the osteogenic differentiation program.

Our finding that miR-378 is strongly upregulated during C2C12 myogenic differentiation corresponds well to other reports demonstrating miR-378 upregulation during myogenesis and high levels of this miRNA in skeletal muscle [29,30]. This upregulation of mature miR-378 matches an increase in Pol-II occupancy at a region located within the first intron of the *Ppargc1b* gene, just upstream of the miR-378 gene. This Pol-II enriched area lies adjacent to an E-box containing Myod-binding region previously shown to be important for miR-378 upregulation during myogenesis [29]. Approximately a third of all miRNA genes, including miR-378, lie within introns of protein-coding genes. Such intronic miRNA genes are usually co-regulated with their host genes and subsequently processed to mature miRNAs after splicing of the pre-messenger RNAs [31]. However, the mRNA expression profile of the miR-378 host gene, *Ppargc1b*, as assessed by our microarray analysis, does not fully correspond to the mature miR-378 expression profile; while miR-378 is upregulated during myogenesis, *Ppargc1b* mRNA levels do not change (data not shown). Together with the increase in Pol-II and Myod occupancy seen at sites within the first *Ppargc1b* intron, this might suggest that miR-378 is regulated independently from *Ppargc1b* and transcribed as an independent transcript, an interesting hypothesis that requires further study.

The upregulation of miR-378 specifically during C2C12 myogenic differentiation suggests a role for this miRNA in this pathway. Indeed, a study by Gagan *et al.* has shown that miR-378 promotes C2C12 myogenesis by targeting *Msc* (musculin, also known as myogenic repressor; *MyoR*), a repressor of myogenic differentiation that inhibits Myod activity by binding to its co-activators or binding directly to Myod target sequences [29]. In addition, miR-378 has been shown to target mitogen-activated protein kinase 1 (*Mapk1*) and *Bmp2*, which are relevant to myoblast proliferation and differentiation, respectively, in pigs [30]. Similarly, miR-378 has also been shown to play a role in the repression of cardiac hypertrophy by targeting *Mapk1*, *Igf1r* (insulin-like growth factor 1 receptor), *Grb2* (growth factor receptor-bound protein 2) and *Ksr1* (kinase suppressor of ras 1), components of the MAP kinase pathway, in rat cardiomyocytes [32]. In contrast, we did not observe any significant effect of overexpression of miR-378 on C2C12 myogenesis, as assessed by the expression of several myogenic marker genes and Ck activity. The discrepancy with the work of Gagan *et al.* might be attributed to a difference in levels of miR-378 overexpression resulting from the use of different overexpression methods (transient lipofectamine transfection versus our stable lentiviral-transduced cell lines). Alternatively, since the positive effects on myogenesis seen by Gagan *et al.* were at early time points (day 1 and day 3), it is possible that overexpression of miR-378 merely accelerates myogenesis and similar maximal levels have been reached by both miR-378 overexpressing and control cells at the later time points that we investigated (day 3 and day 6). Our observation that miR-378 overexpression has no apparent effect on myogenesis does not rule out that it plays a role in this process; most likely, endogenous levels of this miRNA are sufficient for its biological function, and overexpression has no additional effect on myogenic markers.

It would, however, still be interesting to take a closer look at the genes that are downregulated by miR-378 overexpression in undifferentiated myoblasts (day 0 time point); genes that are downregulated during C2C12 myogenesis, and significantly downregulated by miR-378 overexpression in myoblasts, such as for example (data not shown) *Fgf7* (fibroblast growth factor 7), *Crlf1* (cytokine receptor-like factor 1), *Ereg* (epiregulin) and *Cck* (cholecystokinin), are potential targets of this miRNA and interesting candidates for further study on the role of miR-378 in myogenesis. Unfortunately, we did not observe a significant effect of miR-378 overexpression on mRNA levels of its published targets *Msc*, *Mapk1*, *Igf1r*, *Grb2* and *Ksr1*[29,30,32]. This does not contradict the findings in these publications, since it is possible that miR-378 exerts its effect on these targets at the level of protein translation and not by inducing mRNA degradation (see below).

Besides its putative role in myogenesis, we clearly demonstrate an effect of miR-378 on C2C12 bone differentiation. Our observation that miR-378 overexpression promotes C2C12 osteogenesis in the presence of BMP2, as assessed by Alp activity, calcium deposition and expression of osteogenic marker genes, was surprising considering the lack of changes in its expression level during BMP2-induced osteogenic differentiation. Since this effect of miR-378 overexpression is limited only to BMP2-treated cells, we believe that miR-378 on its own is not a major determinant of the osteogenic cell fate, but more likely plays a role in fine-tuning osteogenic gene expression within the BMP2-induced cellular environment.

A role for miR-378 in modulating osteogenic differentiation has previously been described by Kahai *et al.* in the context of a nephronectin (*Npnt*)-3’UTR overexpressing MC3T3-E1 osteo-progenitor cell line [33]. Npnt is an extracellular matrix protein that, when overexpressed, enhances MC3T3-E1 osteoblast differentiation. Npnt secretion depends on its glycosylation by glycosylation-associated enzymes including Galnt7 (UDP-N-acetyl-alpha-D-galactosamine:polypeptide N-acetylgalactosaminyltransferase 7). The 3’UTR of both *Npnt* and *Galnt7* contain a miR-378 binding site. Kahai *et al.* demonstrated that, during late stages of MC3T3-E1 development (in the presence of ascorbic acid, β-glycerophosphate and dexamethasone), stable cell lines overexpressing *Npnt* containing its 3’UTR (*Npnt-3’UTR*) have a higher rate of osteoblast differentiation and bone nodule formation than cell lines overexpressing *Npnt* without its 3’UTR; this is further enhanced by co-transfection with *miR-378*. Interestingly, co-transfection of *Npnt-3’UTR* with *miR-378* enhanced production of Npnt and promoted Npnt glycosylation. It was suggested that interaction of the *Npnt* 3’UTR with miR-378 sequestered this miRNA away from *Galnt7*, leading to enhanced Galnt7 activity, a subsequent increase in Npnt glycosylation and secretion and, as a result, a higher rate of osteogenesis. In addition, it was proposed that binding of miR-378 to the *Npnt* 3’UTR resulted in preventing access of other miRNAs, thereby protecting the *Npnt* mRNA from post-transcriptional regulation and resulting in the observed increase in Npnt synthesis [33]. In line with these findings, we observed significantly higher levels of *Npnt* mRNA in our C2C12-pMirn378 versus control cells after 6 days of osteogenic differentiation (data not shown). It would therefore be interesting to determine whether a similar Npnt/Galnt7 –mediated mechanism might also play a role in the effect miR-378 overexpression has on BMP2-induced C2C12 osteogenesis. However, the positive effect of miR-378 overexpression on MC3T3-E1 osteoblast differentiation described by Kahai *et al.* was only observed when co-transfected with *Npnt-3’UTR* and only during later stages of development. In fact, stable transfection of MC3T3-E1 cells with *miR-378* alone actually inhibited osteogenesis [33]. This is in direct contrast with our observation in BMP2-induced C2C12 cells and indicates that the effect of miR-378 may depend on the osteogenic model system used and/or the signaling pathways involved in inducing differentiation: for example, it is conceivable that miR-378 acts specifically on the BMP2 signaling pathway to positively reinforce the BMP2 effect on our C2C12 model system, while this mechanism might not play a role in the differentiation of MC3T3-E1 cells by Kahai *et al.,* which occurred in the absence of BMP2. Further exploration of the mechanism underlying the positive effect of miR-378 on our BMP2-induced C2C12 system may help shed light on this issue.

We were as yet unable to identify the genes that are directly targeted by miR-378 during BMP2-induced C2C12 osteogenesis. Most effects seen in our mRNA microarray analysis are likely to be secondary to the initial effect of miR-378, making it difficult to identify its direct target(s). Given the overall positive effect of miR-378 on the expression of osteogenic markers, and negative effect on myogenic markers, we expected the initial targeting event to take place early during the differentiation process. To identify direct miR-378 targets, we therefore selected genes *a)* that were downregulated by miR-378 overexpression early (day 0) and consistently during osteogenesis, *b)* that contained a predicted miR-378 target site in their 3’UTR and *c)* that were known to play a role in the regulation of osteoblast differentiation. This led to the selection of *Grem1, Wnt5a* and *Wnt10a* as putative targets. Grem1 is a secreted glycoprotein that binds BMP2 and prevents BMP2 signaling and activity in cells of the osteoblast lineage [34]. Targeting of *Grem1* by miR-378 could thus increase the levels of BMP2 available for inducing osteogenesis. Wnts are a family of 19 secreted glycoproteins that activate their cell surface receptors to induce specific intracellular signaling cascades controlling gene expression and play a critical role in embryonic development, postnatal development and adult tissue homeostasis [35]. Wnt signaling regulates cellular processes including proliferation, differentiation, and apoptosis through β-catenin-dependent canonical and β-catenin-independent non-canonical pathways and has been shown to play an important role in bone formation [36]. Wnt5a has been found to be the most dominant Wnt expressed during osteogenesis of human mesenchymal stem cells (hMSCs) both *in vitro* and *in vivo*[37] and Wnt5a signaling has been shown to be important for BMP2-mediated osteogenesis in MC3T3-E1 cells, though the exact signaling pathways involved remain unclear [38]. Wnt10a has also been shown to stimulate osteogenesis [39]. Given their important role in osteoblast formation, it was interesting to determine whether these Wnts were indeed targeted by miR-378 and subsequently how this could relate to the observed increase in osteogenic differentiation. However, our luciferase-reporter assay demonstrated that miR-378 did not directly target the 3’UTR of any of these selected candidates and further work is thus required to identify the mechanism by which miR-378 exerts its effect.

The imperfect complementarity that may exist between a miRNA and its target, the possibility for combinatorial regulation that depends on the presence of other miRNAs to observe an effect, and the various mechanisms by which miRNAs may act, pose a great challenge common to all studies of miRNA function. In our approach we assumed that miR-378 exerts its effect by mRNA destabilization and/or degradation, resulting in a decrease in mRNA levels of its target(s). It is possible, however, for a miRNA to have only very subtle effects on (multiple) targets that cannot be observed as a change in mRNA levels of its direct targets, or to affect protein translation without affecting mRNA levels [14,40]. In addition, miRNAs have been shown to be able to affect mRNA levels of their target genes via alternative mechanisms than binding to their 3’UTR, which would not be detected using a luciferase-3’UTR reporter assay. For instance, it has been shown that miRNAs can affect gene transcription by inducing histone modifications at target promoter sites [41]. Interestingly, a study by Gerin *et al.* has shown that miR-378 can specifically increase the transcriptional activity of Cebpa and Cebpb (CCAAT/enhancer binding protein, alpha and beta) on adipocyte gene promoters, though it could not be excluded that this was an indirect effect through e.g. inhibition of a co-repressor [42]. Given the role of Cebpb in synergizing with Runx2 to regulate bone-specific gene expression [43], it would be very interesting to investigate whether a similar mechanism underlies the effect of miR-378 on BMP2-induced osteogenesis.

So far, we have attributed the effects seen in C2C12 cells transduced with the miR-378 precursor expression construct to mature miR-378, the 3p strand of the precursor miRNA. However, it should be noted that these cells also overexpress miR-378*, the less-abundant 5p strand. Although present at 10–30 times lower levels than miR-378 (data not shown), it cannot be excluded that the effects seen are (in part) the result of miR-378* overexpression, and it would thus be interesting to also search for putative miR-378* targets within the group of affected genes.

In this study, we used our previous Pol-II ChIP-on-chip dataset to identify lineage-specific miRNA expression. Since the probes on the arrays used for this dataset were restricted to promoter sequences of protein coding genes, the results of this approach do not represent the full picture of Pol-II occupancy at all miRNA gene promoters in the genome. This could explain why several miRNAs known to be specifically upregulated during myogenesis, the so-called myomiRs (*miR-1/206* and *miR-133* families) [12], were not identified. However, our approach did provide a first means to identify several miRNAs with differential Pol-II occupancy during myogenic versus osteogenic differentiation. Most of these miRNAs, including miR-21, miR-34b/c, miR-99b, miR-365 and miR-675, have an as yet unknown role in these differentiation pathways and are thus attractive candidates for further investigation.

## Conclusions

In the present study we have identified a list of miRNAs that potentially play a role in C2C12 lineage specification and demonstrated a previously unknown role for miR-378 in enhancing BMP2-induced osteogenic differentiation. Future studies will focus on further exploring the precise function of these miRNAs during cellular differentiation, including the challenging task of identifying their targets and mechanisms of action.

## Methods

### Cell culture and treatment

Murine C2C12 myoblasts (as well as C2C12-derived stable cell lines: see below) and Human Embryonic Kidney (HEK) 293 cells (American Type Culture Collection, Manassas, VA) were maintained at subconfluent densities in DMEM (Invitrogen, Carlsbad, CA), supplemented with 10% newborn calf serum (NCS; Thermo Fisher Scientific, Waltham, MA), antibiotics (100 U/ml penicillin, 100 μg/ml streptomycin: Sigma-Aldrich, St. Louis, MO), and 2 mM L-glutamine (Invitrogen), further designated as growth medium (GM), at 37°C in a humidified atmosphere containing 7.5% CO_2_. To study C2C12 differentiation, cells were plated at 2.5 × 10^4^ cells per cm^2^ in GM and grown for 24 hours to sub-confluence. Subsequently, medium was replaced by DMEM containing 5% NCS (referred to as differentiation medium (DM)) in the presence or absence of 300 ng/ml recombinant human bone morphogenetic protein 2 (BMP2; R&D Systems, Minneapolis, MN). For calcium deposition studies, 0.2 mM ascorbate and 10 mM β-glycerophosphate were added to the DM. Medium was replaced every 3–4 days.

### Pol-II ChIP-on-chip and selection of differentially enriched microRNA genes

Generation of the RNA polymerase II (Pol-II) ChIP-on-chip dataset used in this study has been described in Hupkes *et al*. [27]. Enriched regions within 500 base pairs (bp) upstream from a miRNA transcription start site (TSS), within the miRNA gene, or up to 500 bp downstream from the gene end were assigned to that associated miRNA. MiRNA-associated active regions with an absolute average log2 fold > 0.4 of untreated over BMP2-treated Pol-II enrichment values at each time point were selected as differentially expressed during myogenic versus BMP2-induced osteogenic C2C12 differentiation.

### RNA isolation and miRNA real-time polymerase chain reaction (PCR)

RNA (including miRNA) was extracted using TRIzol® according to the manufacturer’s instructions (Invitrogen). RNA was precipitated with isopropanol and, after air-drying, dissolved in DEPC-treated H_2_O. Total RNA concentrations were quantified by measuring absorbance at 260 nm.

The TaqMan® miRNA Reverse Transcription Kit (Applied Biosystems, Carlsbad, CA), including TaqMan® stem-loop primers miR-378 and miR-365 (cat. # 4427975_002243 and 4427975_001020, respectively; TaqMan® miRNA Assays (Applied Biosystems)) and snoRNA202 (cat. # 4427975_001232; TaqMan® Small RNA Control Assays (Applied Biosystems)) were used for reverse transcription (RT) of miR-378 (3p), miR-365 (3p) and the small, non-coding control RNA snoRNA202 from 100 ng of total RNA each, according to the manufacturer’s protocol.

TaqMan® PCR primers and probes for miR-378, miR-365 and snoRNA202, included in the above-mentioned TaqMan® miRNA and small RNA Control assays, together with the TaqMan® Universal PCR Master Mix II, no uracil N-glycosylase (UNG) (Applied Biosystems) were subsequently used for quantitative PCR analysis, also according to the manufacturer’s instructions. MiR-378 and miR-365 expression levels were expressed as a percentage of the control small, non-coding RNA snoRNA202.

### Expression constructs

The lentivector-based miR-378 precursor expression construct PMIRH378PA-1 (referred to as ‘pMirn378’) and its parent lentivector pCDH-CMV-MCS-EF1-copGFP (referred to as ‘pMirn0’) were purchased from System Biosciences (Mountain View, CA). Both vectors contain an expression module for the copGFP fluorescent marker gene to enable monitoring of cells positive for transfection and transduction. MiTarget™ 3’UTR miRNA target clones were purchased from GeneCopoeia (Rockville, MD) and consisted of the *Grem1, Wnt5a* or *Wnt10a* (accession numbers NM_011824.3, NM_009524.2 and NM_009518.1 respectively) 3’UTR sequence, the miR-378 target sequence (5’-CCTTCTGACTCCAAGTCCAGT-3’; positive control) or no additional sequence (negative control) inserted in the pEZX-MT01 vector downstream of the firefly luciferase reporter gene (constructs are referred to as Grem1-luc, Wnt5a-luc, Wnt10a-luc, Pos-luc and Neg-luc, respectively). The firefly luciferase gene, driven by an SV40 promoter, resulted in the transcription of a chimeric transcript consisting of luciferase and the inserted target sequence. The pEZX-MT01 vector also contained the *Renilla* luciferase gene under the control of a CMV promoter to allow dual analysis of firefly and *Renilla* luciferase activities in individual samples. Firefly luciferase activity was thus normalized to account for potential differences in transfection efficiencies between different samples.

### Stable C2C12-pMirn cell lines

Lentiviruses were produced from pMirn378 and pMirn0 as described previously [44]. For infection, C2C12 cells were initially seeded in a 24-wells plate in GM at a density of 3.0 × 10^3^ cells per well. The next day, cells were infected for 48 hours with 800 ng of virus in GM containing 8 μg/ml of polybrene, whereby the infection medium was refreshed after 24 hours. The cells were then washed twice with GM and maintained in GM for another 24 hours. Subsequently, cells were transferred to T75 flasks and maintained in GM until a confluency of approximately 60% was reached. Finally, copGFP-positive cells were sorted by FACS, resulting in the stably transduced cell lines C2C12-pMirn0 and C2C12-pMirn378.

### Microarray processing and identification of significantly regulated genes

For mRNA expression profiling analysis, total RNA samples were purified using the RNeasy Mini Kit (Qiagen, Venlo, the Netherlands), according to the manufacturer’s RNA cleanup protocol. Quality of RNA samples was evaluated by capillary electrophoresis on an Agilent 2100 Bioanalyzer (Agilent Technologies, Santa Clara, CA). In total, 30 RNA samples were obtained from triplicate experiments of C2C12-pMirn0 or C2C12-pMirn378 cells cultured for 0, 3 or 6 days in DM with or without 300 ng/ml BMP2. Following purification, 200 ng of total RNA were amplified, labeled, and fragmented using the GeneChip 3’ IVT Express Kit (Affymetrix, Santa Clara, CA) according to the manufacturer’s instructions. Fragmented amplified RNA (10 μg) was subsequently applied to the GeneChip Mouse Genome 430 2.0 array (Affymetrix) and hybridized for 16 hours at 45°C at 60 rpm in a GeneChip Hybridization Oven 640 (Affymetrix). Following hybridization, the arrays were washed and stained with a GeneChip Fluidics Station 450 (Affymetrix) using the Affymetrix Hybridization Wash Stain (HWS) kit. The arrays were laser scanned with a GeneChip Scanner 3000 7G (Affymetrix). Data was saved as raw image file and quantified using Affymetrix GeneChip Command Console v 1.0 (Affymetrix). These data were imported to R 2.4.1 using the Bioconductor (http://www.bioconductor.org) Affymetrix package. The model-based Robust Multiarray Average (RMA) algorithm was used to generate the probe set summary based on the full annotation on gene level and normalization was done according to the quantile method. To identify genes that are differentially expressed in C2C12-pMirn378 versus C2C12-pMirn0 samples, expression ratios were calculated for each time point and treatment using the Limma algorithm in R, applying moderated t-tests. A similar approach was taken to identify genes that are up- or downregulated during differentiation of C2C12-pMirn0 cells, whereby expression ratios were calculated for each time point during each treatment versus the d0 base line value. To correct for multiple hypothesis testing, the q value [45] was calculated for each p value using Benjamini-Hochberg correction, indicating the significance of the corresponding ratio.

Genes with a q value < 0.005 and an absolute log2 expression ratio between C2C12-pMirn378 and C2C12-pMirn0 > 0.6 were considered to be significantly differentially expressed at the corresponding time point and treatment. Genes with a q value < 0.005 for the d6 vs d0 time point and an average log2 expression ratio of the d3 vs d0 and d6 vs d0 time points < −0.6 or > 0.6 for the same treatment were considered to be significantly down- or upregulated, respectively, during that particular treatment. Results are listed in Additional file 2. In addition, raw and processed microarray data were submitted to the U.S. National Center for Biotechnology Information Gene Expression Omnibus (GEO) database (GSE51883). The Web-based platform DAVID Bioinformatics Resources [46] was used to identify enriched Gene Ontology (GO) terms of the biological process category [47] in the sets of significantly differentially expressed genes relative to all probes represented on the array, whereby a p value < 0.01 was considered a significant enrichment.

### Target prediction

TargetScan version 4.0, PITA, DIANA, PicTar, FINDTAR3 and Miranda databases were used to identify potential miR-378 target sites in genes that were downregulated in C2C12-pMirn378 cells as compared to C2C12-pMirn0 cells.

### Transfections and luciferase reporter assays

HEK293 cells were seeded in 24-well plates in GM and medium was refreshed after 24 hours. One hour prior to transfection, medium was replaced by GM lacking antibiotics. 3’UTR miRNA target clones (0.4 μg) were subsequently co-transfected with pMirn0 or pMirn378 (0.4 μg) using Lipofectamine 2000 (Invitrogen) according to the manufacturer’s instructions. After 5 hours of incubation with transfection reagents, medium was replaced by GM. Twenty-four hours later, firefly and *Renilla* luciferase activities were measured from the same samples using the LucPair™ miR Duo-Luciferase Assay Kit according to the manufacturer’s instructions (Genecopoeia). Firefly luciferase activity was then normalized for transfection efficiency using the *Renilla* luciferase activity in the same sample. Normalized luciferase values are presented as percentage of the control samples co-transfected with the Neg-luc vector.

### Creatine kinase assay

Creatine kinase (Ck) enzymatic activity was measured in cell lysates using the EnzyChrom™ Creatine Kinase Assay Kit (ECPK-100, BioAssay Systems, Hayward, CA) according to the manufacturer’s protocol. Cell lysates were obtained from cells seeded in 48-well plates: cells were washed twice with PBS, lysed by incubation in 50 μl lysis buffer (Promega, Madison, WI) on ice for 10 minutes, scraped loose and spun down to remove cellular debris. Supernatant was then collected and diluted 2.5 times in H_2_O, of which 10 μl was used for each Ck measurement. Results of the Ck assay were normalized for protein content, as measured using the Bio-Rad Protein assay (Bio-Rad, Hercules, CA) according to the manufacturer’s protocol (“Microassay Procedure for Microtiter Plates”) and thus expressed as ‘arbitrary units (AU)’. Samples were diluted such that absorbance at 595 nm for each sample fell within the linear range of a bovine serum albumin (BSA) standard curve.

### Alkaline phosphatase and mineralization assays

Alkaline phosphatase (Alp) enzymatic activity was measured as described previously [48] and normalized for neutral red staining to correct for potential differences in cell number [49].

Calcium deposition in the extracellular matrix (calcium release) was measured as described by Piek *et al.*[44].

### Statistical analysis

For miRNA real-time PCR analysis, Ck, Alp, calcium and luciferase assays, Student’s 2-tailed *t* test was used to compare miR-378-overexpressing samples with their controls whereby a difference with p < 0.05 was considered significant.

### Availability of supporting data

The raw and processed microarray data sets supporting the results of this article are available in the NCBI GEO repository, GSE51883, http://www.ncbi.nlm.nih.gov/geo/query/acc.cgi?acc=GSE51883.

## Competing interests

The authors declare that they have no competing interests.

## Authors’ contributions

MH conceived of the study, participated in its design and coordination, carried out part of the molecular and cellular studies and drafted the manuscript. AS participated in the design, carried out the luciferase assays, helped to draft the manuscript and performed the statistical analysis. JH carried out the miRNA qPCR studies. JvZ and KD participated in the design and coordination of the study and helped to draft the manuscript. All authors read and approved the final manuscript.

## Supplementary Material

Additional file 1: Figure S1 Microarray expression profiles of control C2C12-pMirn0 cells. mRNA expression profiles of A) the muscle transcription factors myogenin (*Myog;* 1419391_at) and myocyte enhancer factor 2C (*Mef2c;* 1421027_a_at) and other muscle marker genes muscle creatine kinase (*Ckm;* 1417614_at), the acetylcholine receptor subunit gamma (*Chrng;* 1449532_at) and the sarcomeric genes fast troponin C2 (*Tnnc2;* 1417464_at) and actinin alpha 3 (*Actn3;* 1418677_at), B) the osteogenic transcription factors Sp7 transcription factor 7 (*Sp7;* 1418425_at), distal-less homeobox 5 (*Dlx5;* 1449863_a_at) and runt-related transcription factor 2 (*Runx2;* 1424704_at), and other osteogenic marker genes alkaline phosphatase (*Alpl;* 1423611_at), bone gamma-carboxyglutamate (gla) protein (*Bglap;* 1449880_s_at) and vitamin D receptor (*Vdr;* 1418175_at) and C) the cell-cycle regulators cyclins A2 (*Ccna2;* 1417910_at*)* and B1 (*Ccnb1;* 1419943_s_at), cell division cycle 7 (*Cdc7;* 1426002_a_at) and 20 (*Cdc20;* 1416664_at) and the cyclin-dependent kinases 1 (*Cdk1;* 1448314_at) and 4 (*Cdk4;* 1422441_x_at) at indicated time points during differentiation of C2C12-pMirn0 cells treated with (diamonds) or without (circles) 300 ng/ml BMP2 as revealed from microarray analysis. Mean expression values +/− SD from triplicate microarray experiments are shown for all data points. When the error bar is not visible, the SD falls within the printed data point. All SD values are, however, listed in Additional file 2. AEV = average expression value. Click here for file

Additional file 2: Table S1 Results of mRNA expression profiling. Gene expression profiling results, listing normalized values in C2C12-pMirn0 and C2C12-pMirn378 cells after 0 (d0), 3 (d3) and 6 (d6) days of treatment with or without 300 ng/ml BMP2 as average and standard deviation of three biological replicates, including q values for indicated combinations. Genes that are significantly up- or downregulated during myogenic (column AF) and osteogenic (column AG) differentiation of C2C12-pMirn0 control cells are indicated with ‘SU’ and ‘SD’, respectively, in the appropriate columns. Genes that are significantly up (SU)- or downregulated (SD) in C2C12-pMirn378 cells as compared to C2C12-pMirn0 cells during myogenesis (column AH (SD) and AI (SU)) or osteogenesis (column AJ (SD) and AK (SU)) are grouped into 7 groups; 1-significant difference only on d0; 2-significant difference only on d3; 3-significant difference only on d6; 4-significant difference on d0 and d3; 5-significant difference on d3 and d6; 6-significant difference on d0 and d6; 7-significant difference on d0, d3 and d6. Click here for file
