# Supplementary figures and images for: MicroRNA miR-378 promotes BMP2-induced osteogenic differentiation of mesenchymal progenitor cells
Source: BMC Mol Biol. 2014 Jan 27;15:1. doi: 10.1186/1471-2199-15-1 (PMC3905160; doi:10.1186/1471-2199-15-1)

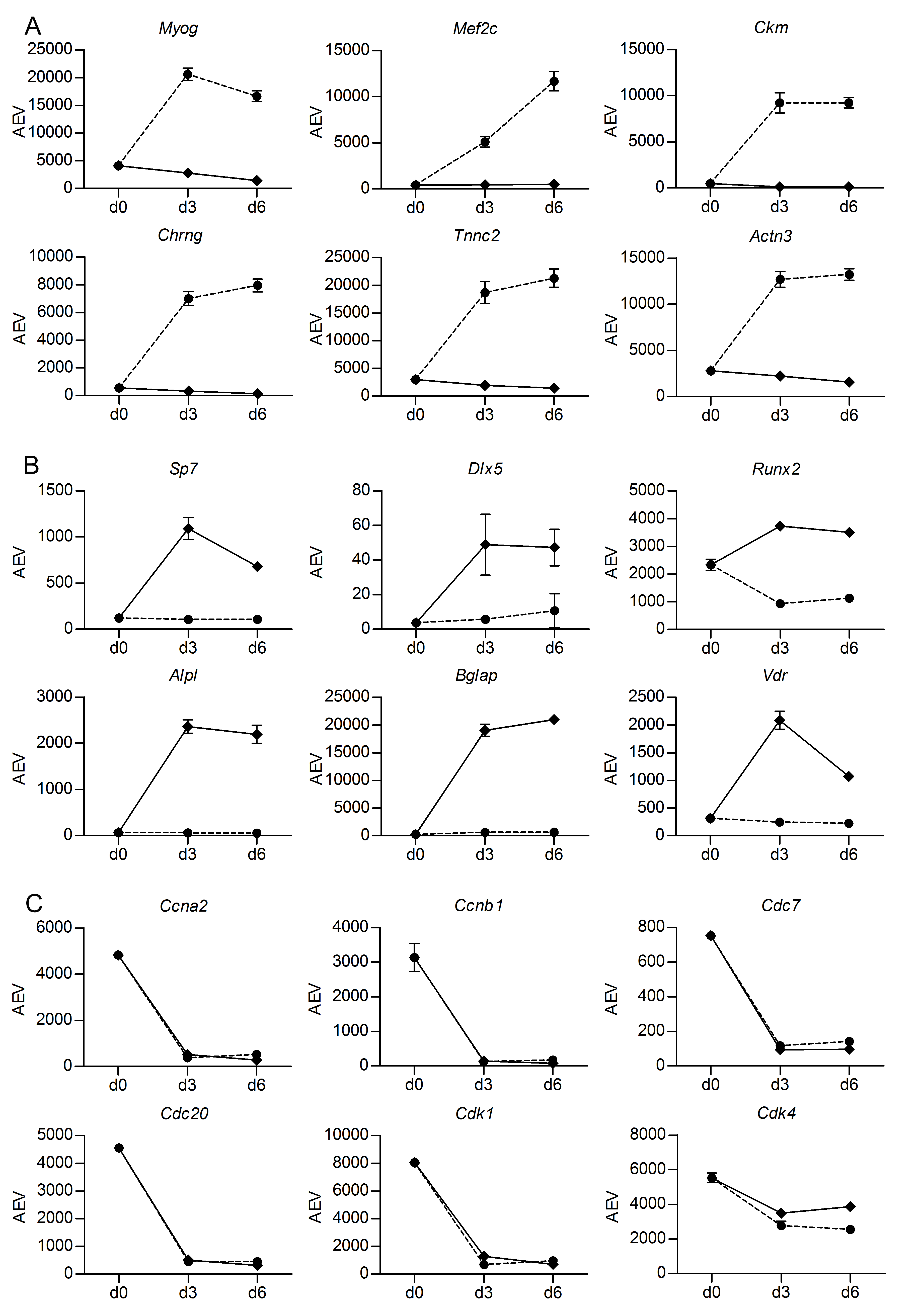

Supplement: Additional file 1: Figure S1 — Microarray expression profiles of control C2C12-pMirn0 cells. mRNA expression profiles of A) the muscle transcription factors myogenin (Myog; 1419391_at) and myocyte enhancer factor 2C (Mef2c; 1421027_a_at) and other muscle marker genes muscle creatine kinase (Ckm; 1417614_at), the acetylcholine receptor subunit gamma (Chrng; 1449532_at) and the sarcomeric genes fast troponin C2 (Tnnc2; 1417464_at) and actinin alpha 3 (Actn3; 1418677_at), B) the osteogenic transcription factors Sp7 transcription factor 7 (Sp7; 1418425_at), distal-less homeobox 5 (Dlx5; 1449863_a_at) and runt-related transcription factor 2 (Runx2; 1424704_at), and other osteogenic marker genes alkaline phosphatase (Alpl; 1423611_at), bone gamma-carboxyglutamate (gla) protein (Bglap; 1449880_s_at) and vitamin D receptor (Vdr; 1418175_at) and C) the cell-cycle regulators cyclins A2 (Ccna2; 1417910_at) and B1 (Ccnb1; 1419943_s_at), cell division cycle 7 (Cdc7; 1426002_a_at) and 20 (Cdc20; 1416664_at) and the cyclin-dependent kinases 1 (Cdk1; 1448314_at) and 4 (Cdk4; 1422441_x_at) at indicated time points during differentiation of C2C12-pMirn0 cells treated with (diamonds) or without (circles) 300 ng/ml BMP2 as revealed from microarray analysis. Mean expression values +/− SD from triplicate microarray experiments are shown for all data points. When the error bar is not visible, the SD falls within the printed data point. All SD values are, however, listed in Additional file 2. AEV = average expression value. [file 1471-2199-15-1-S1.tiff]
